# Supplementary material for: Role and mechanism of NCAPD3 in promoting malignant behaviors in gastric cancer
Source: Front Pharmacol. 2024 Apr 22;15:1341039. doi: 10.3389/fphar.2024.1341039 (PMC11070777; doi:10.3389/fphar.2024.1341039)
Supplement: Supplementary file 11 [file DataSheet2.ZIP › GSEA/immunologic/my_analysis.Gsea.1599462003817/GSE26030_UNSTIM_VS_RESTIM_TH17_DAY15_POST_POLARIZATION_UP.html]

Details for gene set GSE26030\_UNSTIM\_VS\_RESTIM\_TH17\_DAY15\_POST\_POLARIZATION\_UP[GSEA]

|  || Dataset | filtered\_dataset.sample\_info.cls#WT\_versus\_NCAPD3\_MUT |
| Phenotype | sample\_info.cls#WT\_versus\_NCAPD3\_MUT |
| Upregulated in class | WT |
| GeneSet | GSE26030\_UNSTIM\_VS\_RESTIM\_TH17\_DAY15\_POST\_POLARIZATION\_UP |
| Enrichment Score (ES) | 0.46579787 |
| Normalized Enrichment Score (NES) | 1.7561893 |
| Nominal p-value | 0.011824325 |
| FDR q-value | 1.0 |
| FWER p-Value | 1.0 |
Table: GSEA Results Summary

  

Fig 1: Enrichment plot: GSE26030\_UNSTIM\_VS\_RESTIM\_TH17\_DAY15\_POST\_POLARIZATION\_UP      
 Profile of the Running ES Score & Positions of GeneSet Members on the Rank Ordered List

  

| SYMBOL | TITLE | RANK IN GENE LIST | RANK METRIC SCORE | RUNNING ES | CORE ENRICHMENT || 1 | 54894 | RNF43 | 29 | 1.000 | 0.0982 | Yes |
| 2 | 163882 | CNST | 140 | 0.757 | 0.1094 | Yes |
| 3 | 55240 | STEAP3 | 169 | 0.710 | 0.1738 | Yes |
| 4 | 29940 | DSE | 174 | 0.703 | 0.2546 | Yes |
| 5 | 145389 | SLC38A6 | 202 | 0.673 | 0.3153 | Yes |
| 6 | 23216 | TBC1D1 | 355 | 0.563 | 0.2734 | Yes |
| 7 | 9649 | RALGPS1 | 359 | 0.560 | 0.3379 | Yes |
| 8 | 84542 | KIAA1841 | 382 | 0.547 | 0.3872 | Yes |
| 9 | 53944 | CSNK1G1 | 485 | 0.484 | 0.3717 | Yes |
| 10 | 3479 | IGF1 | 511 | 0.469 | 0.4096 | Yes |
| 11 | 7099 | TLR4 | 541 | 0.451 | 0.4424 | Yes |
| 12 | 10274 | STAG1 | 633 | 0.406 | 0.4255 | Yes |
| 13 | 9949 | AMMECR1 | 650 | 0.400 | 0.4616 | Yes |
| 14 | 4669 | NAGLU | 707 | 0.373 | 0.4658 | Yes |
| 15 | 23412 | COMMD3 | 894 | -0.312 | 0.3696 | No |
Table: GSEA details [plain text format]

  

Fig 2: GSE26030\_UNSTIM\_VS\_RESTIM\_TH17\_DAY15\_POST\_POLARIZATION\_UP      
 Blue-Pink O' Gram in the Space of the Analyzed GeneSet

  

Fig 3: GSE26030\_UNSTIM\_VS\_RESTIM\_TH17\_DAY15\_POST\_POLARIZATION\_UP: Random ES distribution      
 Gene set null distribution of ES for **GSE26030\_UNSTIM\_VS\_RESTIM\_TH17\_DAY15\_POST\_POLARIZATION\_UP**
